# Supplementary material for: The First Five Mitochondrial Genomes for the Family Nidulariaceae Reveal Novel Gene Rearrangements, Intron Dynamics, and Phylogeny of Agaricales
Source: Int J Mol Sci. 2023 Aug 9;24(16):12599. doi: 10.3390/ijms241612599 (PMC10454537; doi:10.3390/ijms241612599)
Supplement: Supplementary file 1 [file ijms-24-12599-s001.zip › Supplementary figure.pdf]

Content

Predicted secondary cloverleaf structures for tRNAs of *Cyathus striatus* 87405................................ 2

Predicted secondary cloverleaf structures for tRNAs of *Cyathus striatus* AH440044........................... 3

Predicted secondary cloverleaf structures for tRNAs of *Cyathus jiayuguanensis* 765.......................... 4

Predicted secondary cloverleaf structures for tRNAs of *Cyathus pallidus* QL1................................... 5

Predicted secondary cloverleaf structures for tRNAs of *Cyathus stercoreus* NPCB004........................ 6

Heterogeneous sequence differentiation of mitochondrial genome based on the four data sets........ 7

Base substitution saturation analysis based on the four datasets, conducted by DAMBE.................... 8

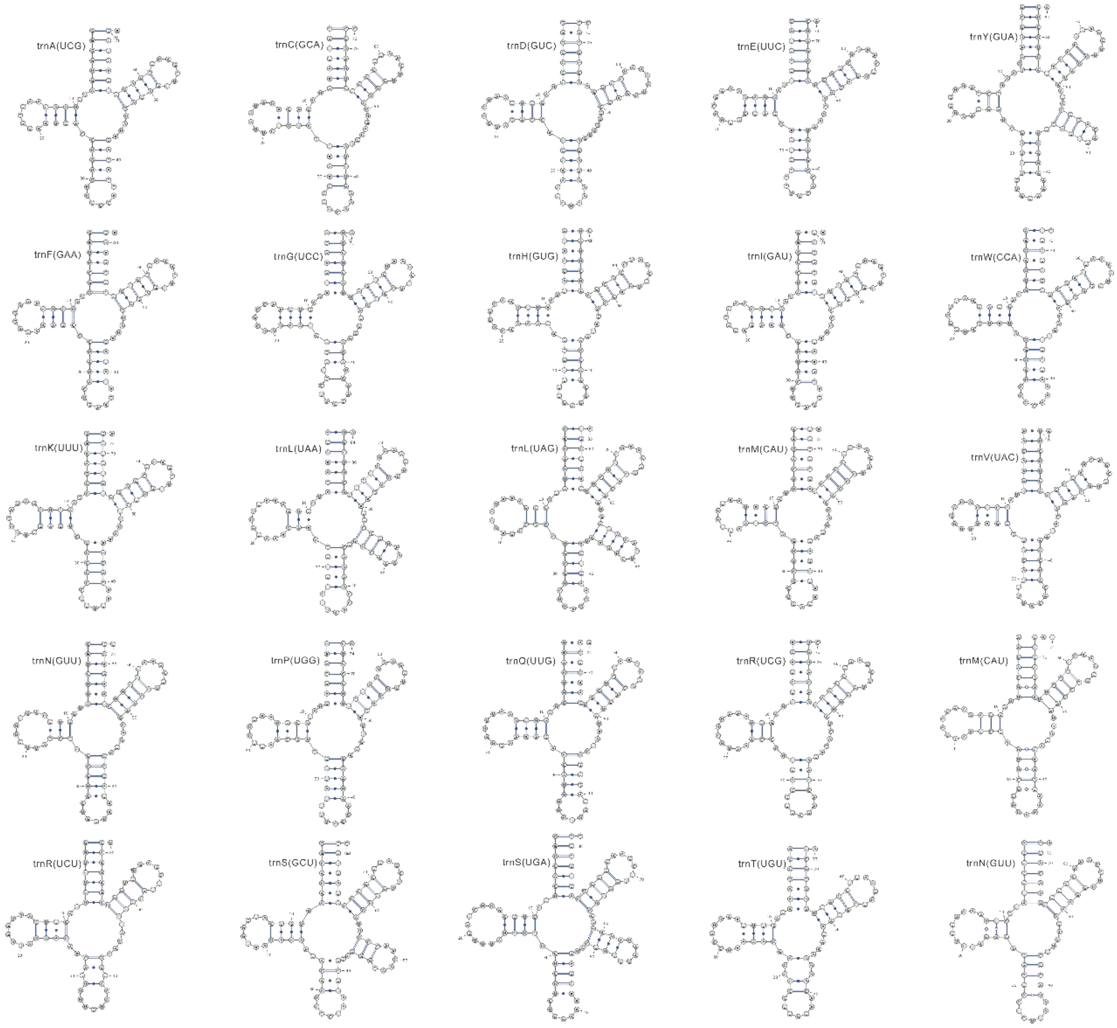

Figure S1. Predicted secondary cloverleaf structures for tRNAs of *Cyathus striatus* 87405.

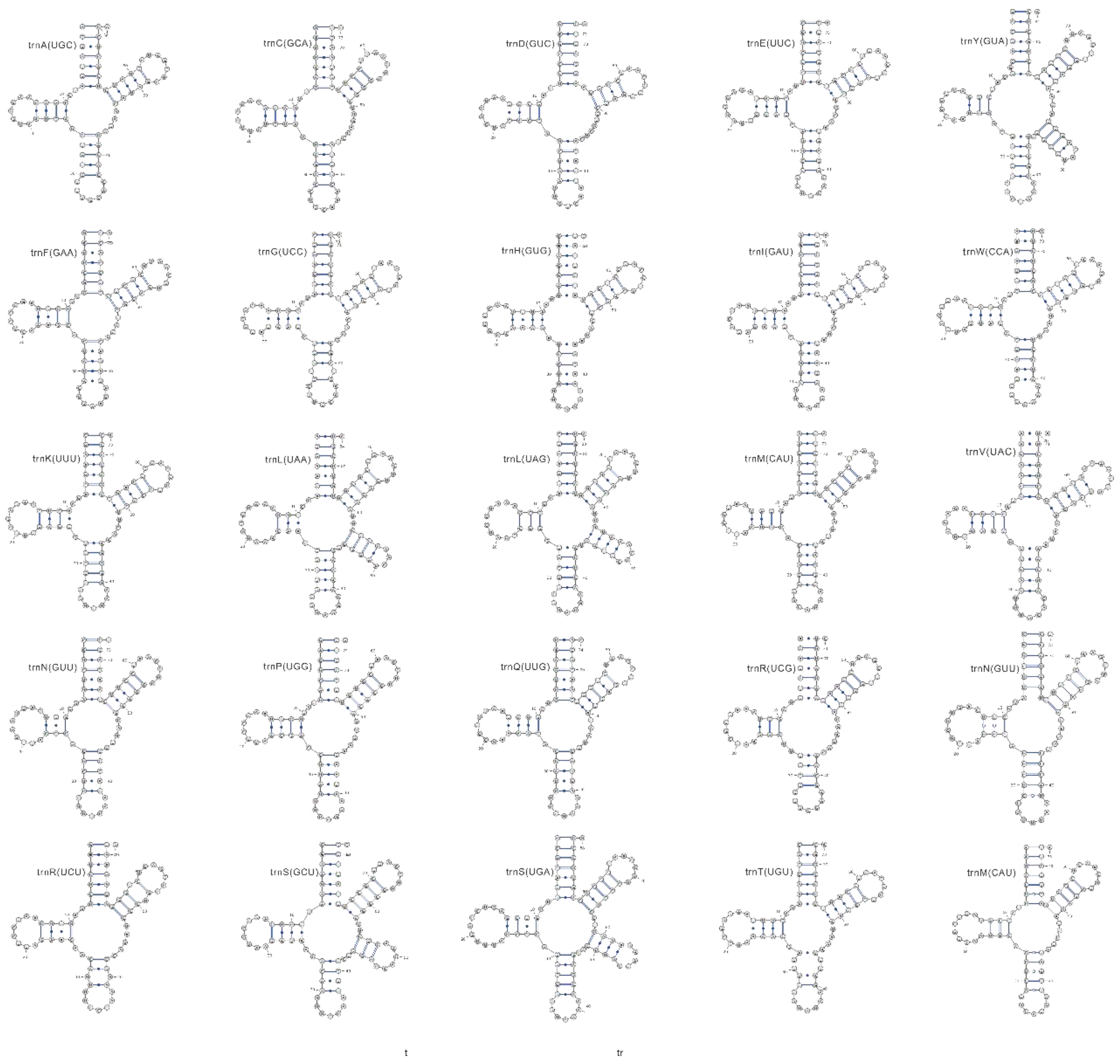

Figure S2. Predicted secondary cloverleaf structures for tRNAs of *Cyathus striatus* AH440044.

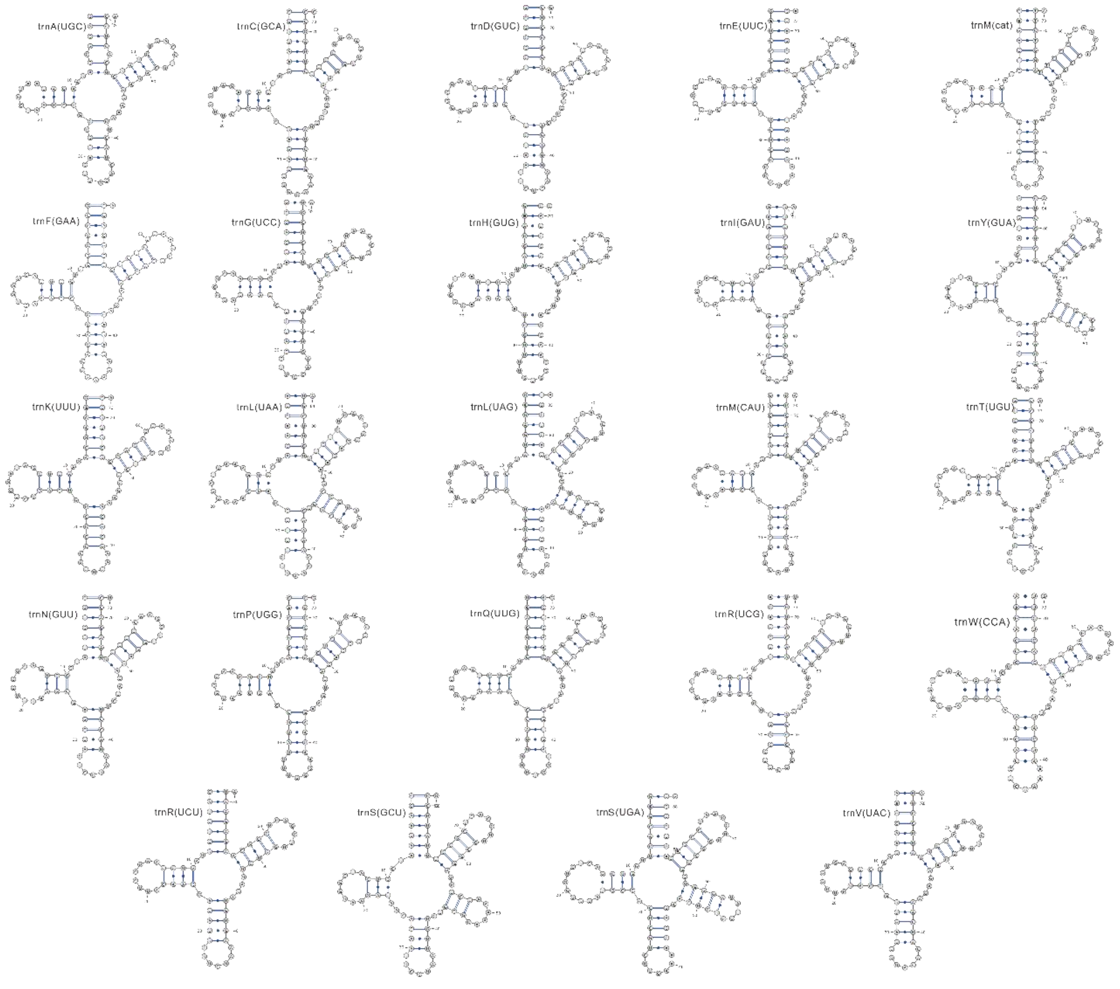

Figure S3. Predicted secondary cloverleaf structures for tRNAs of *Cyathus jiayuguanensis* 765.

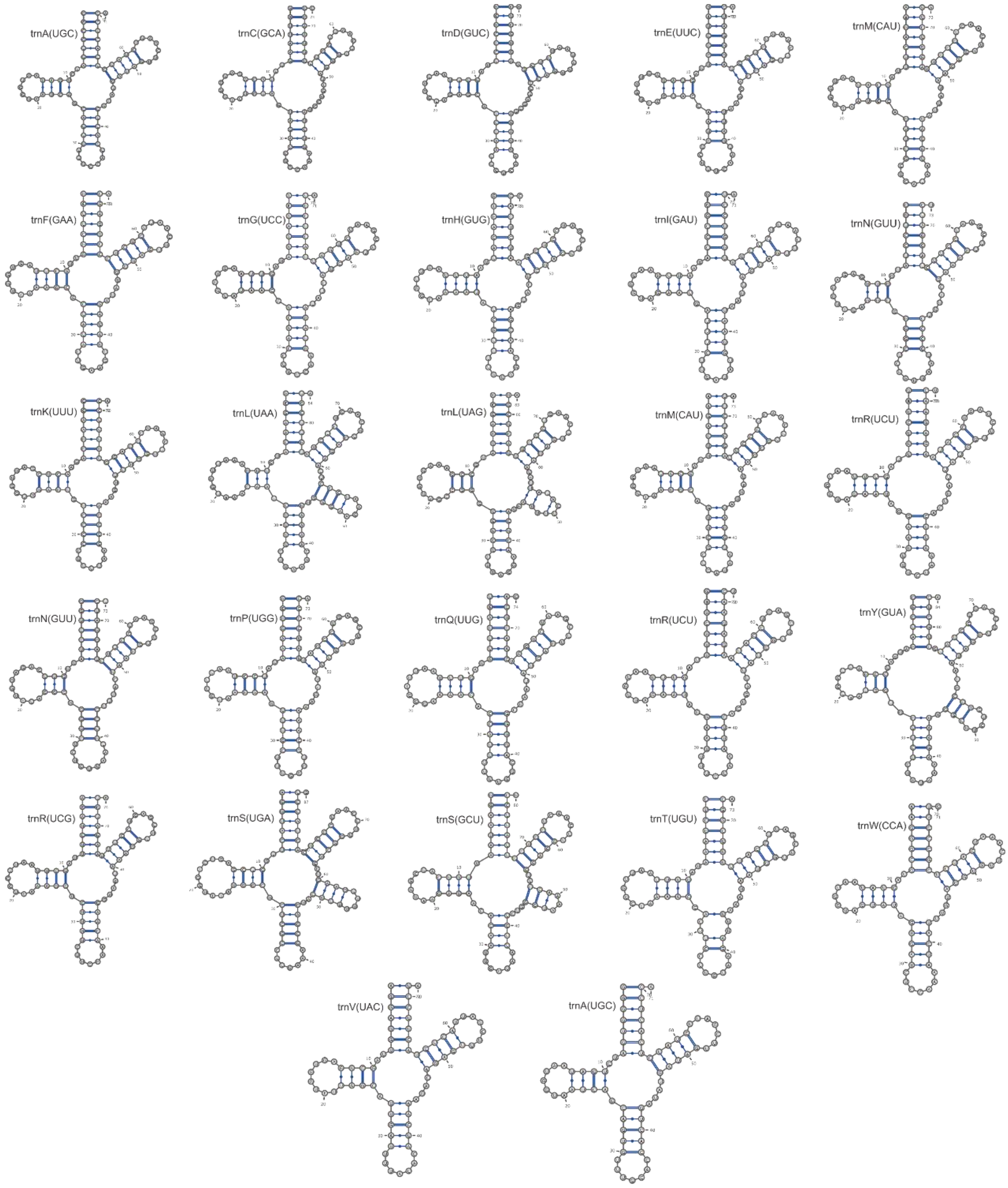

Figure S4. Predicted secondary cloverleaf structures for tRNAs of *Cyathus pallidus* QL1.

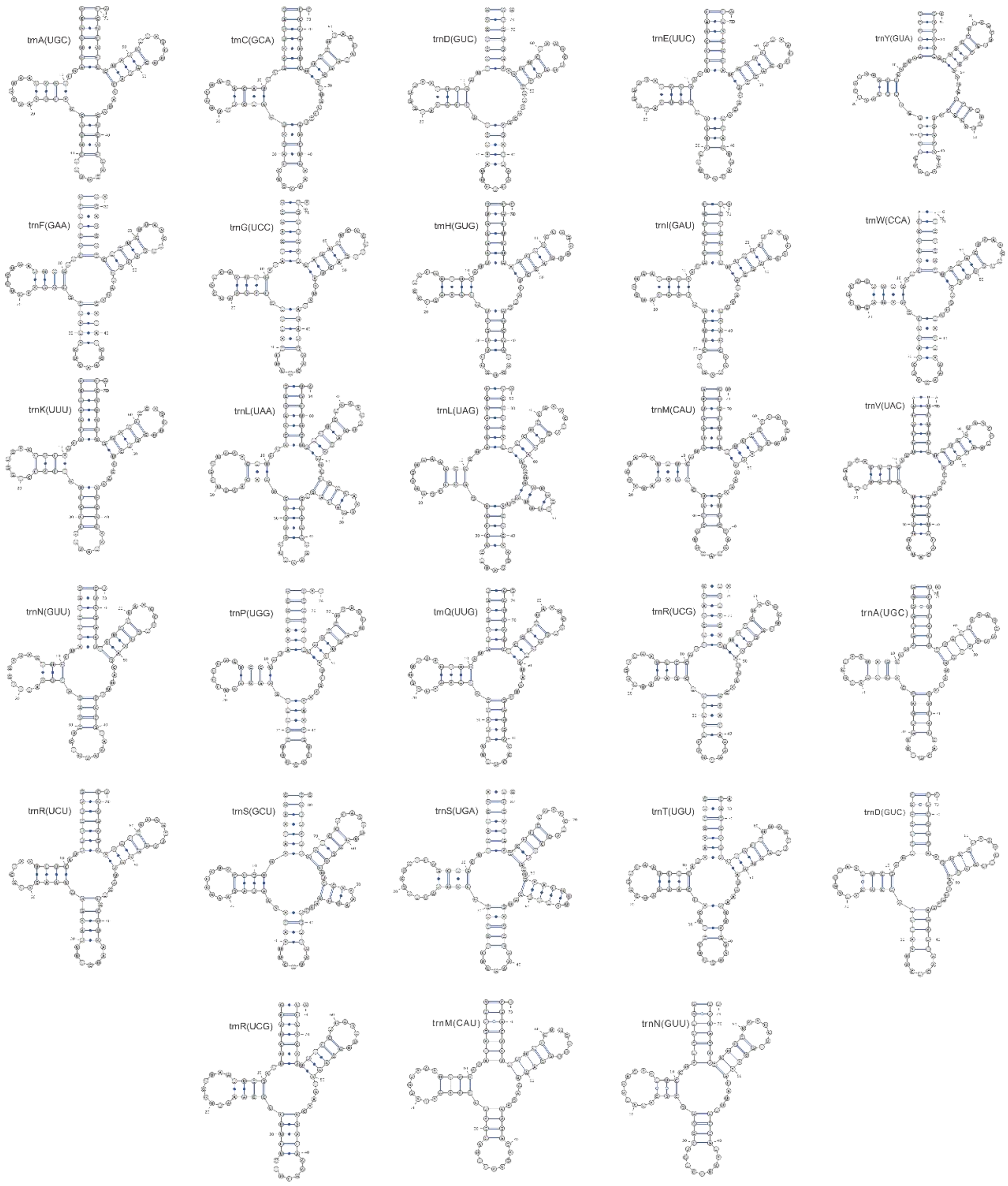

Figure S5. Predicted secondary cloverleaf structures for tRNAs of *Cyathus stercoreus* NPCB004.

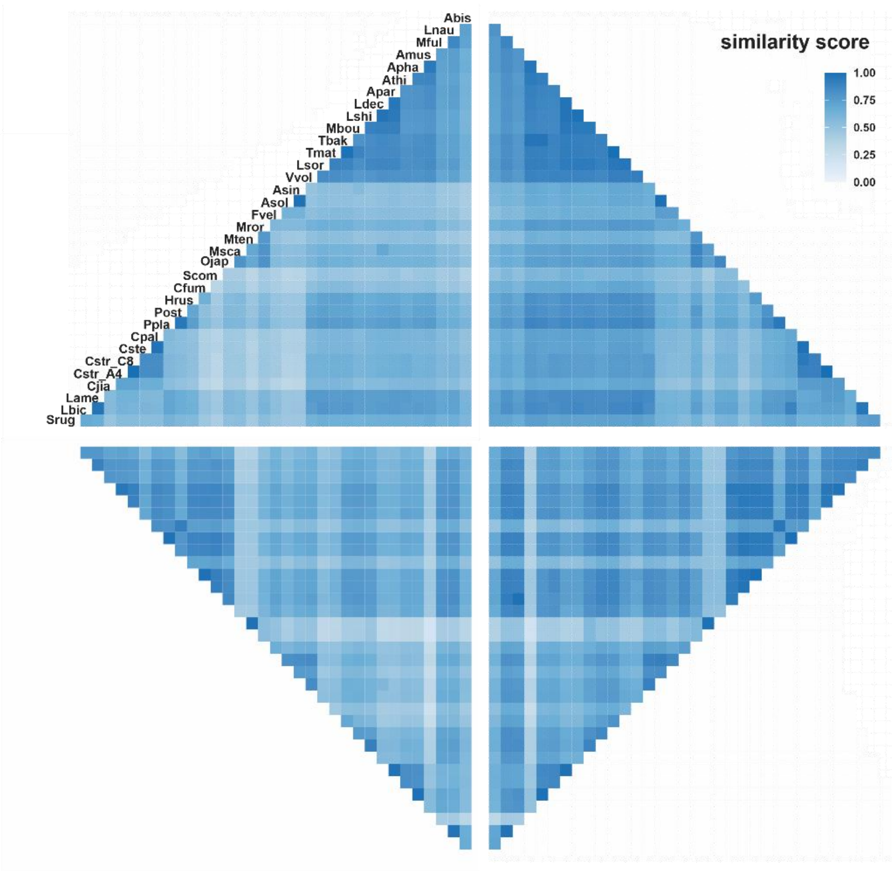

Figure S6. Heterogeneous sequence differentiation of mitochondrial genome based on the four data sets.

The average similarity score between the sequences is represented by a colored block, based on an AliGROOVE2 score from 0 to 1.

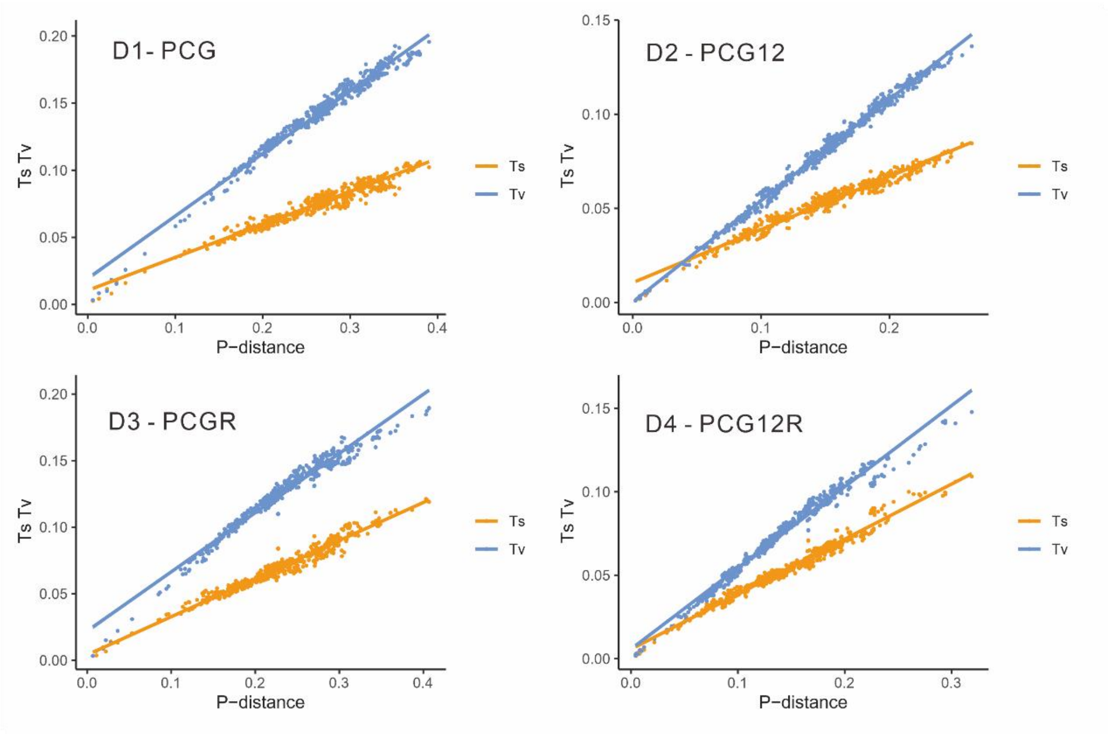

Figure S7. Base substitution saturation analysis based on the four datasets, conducted by DAMBE.
